# Editorial: Novel chimeric antigen receptor (CAR) designs in engineering NK cells for cancer

**DOI:** 10.3389/fimmu.2026.1823184

**Published:** 2026-04-21

**Authors:** Wing Keung Chan

**Affiliations:** Department of Internal Medicine, Division of Hematology, College of Medicine, The Ohio State University, Columbus, OH, United States

**Keywords:** CAR-iNKT cells, CAR-macrophage, CAR-NK cell therapy, car-t, immunotherapy, NK cell, NKG2D (Natural killer group 2 member D), tumor micro environment (TME)

Chimeric antigen receptor (CAR)-based immunotherapy has revolutionized cancer treatment, particular in hematological malignancies such as B cell acute lymphoblastic leukemic and multiple myeloma. Despite highly effective in hematologic malignancies, conventional CAR-T approaches are constrained by unfavorable manufacturing economics, toxicities including cytokine release syndrome and neurotoxicity, limited durable response and modest efficacy in solid tumors. Natural killer (NK) cells, innate cytotoxic lymphocytes capable of killing transformed without prior sensitization and major histocompatibility complex restriction, have emerged as a compelling alternative for CAR engineering. CAR-NK cells promise off-the-shelf availability, lowered toxicity, and intrinsic anti-tumor functions, yet they also present unique designs and translation challenges. This Research Topic brings together 11 original contributions, spanning basic, translational, and clinical perspectives on how we can advance our mechanistic understanding and practical innovation in CAR-NK design, manufacturing, and clinical applications.

We all know NK cells but not T cells intrinsically kill cancer cell. Wang et al.‘s review proposes a conceptual question for the field: “Does a natural killer need a CAR?”. The authors critically examine whether CARs are even necessary for NK function, knowing that NK cells possess inherent cytotoxicity independent of engineered receptors. The authors emphasize that CAR design for NK cells should be specific to NK cel biology, and complement, not override, endogenous receptor networks. Hu et al.‘s systematic review provides a comprehensive overview of the rapid advances in CAR-NK cell therapy, highlighting how genetic engineering has refined CAR structural components, optimized gene delivery strategies, and expanded the repertoire of viable NK cell sources. By summarizing current technologies, cellular platforms, and engineering approaches, it underscores both the remarkable preclinical progress and persistent challenges such as limited *in vivo* persistence, manufacturing scalability, and immunosuppressive tumor microenvironments (TME). All these must be overcome for broader clinical application.

## Redesigning CAR modules for NK cell-specific signaling

One common theme across these contributions is the push beyond T-cell-derived CAR architectures toward functional modules optimized for NK cell biology. T-cell based CARs incorporate intracellular co-stimulation domains such as CD3ζ with co-stimulation motifs (4-1BB, and CD2) optimized for T cell functions. However, NK cells utilize distinct activation pathways such as NKG2D, 2B4 (CD244), DAP12, and SLAM family adaptors. dos Santos et al. demonstrated that CAR constructs incorporating 2B4 and DAP12 co-stimulation domains into an anti-CD19 CAR enhance NK cell specific activation compared to classical 4-1BB designs. Using the NK-92 cell line as a controlled platform, the authors demonstrated superior cytotoxic transcriptional gene signatures, and *in vivo* tumor control together with transient modulation with dasatinib, outperforming conventional 4-1BB-CD3ζ CAR constructs. These findings underscore the importance of co-stimulatory domain selection that aligns with NK cell signaling biology. This concept of using NK cell-specific CAR is further supported by a review article from Han et al. The authors review CAR designs based on the blueprint of the NK activation receptor NKG2D and leverage its stress-induced ligands, which are widely expressed on tumors including both hematological malignancies and solid tumors. The review also highlights engineering innovations such as multi-specific targeting, optimized signaling domains, and strategies to overcome TME suppression, while also addressing challenges including ligand shedding, manufacturing fratricide, and potential on-target off-tumor effects.

Another contribution explores non-scFv extracellular targeting domain as a strategy to expand CAR design space and enhance tumor specificity and ligand affinity. He et al. reported using HER2-specific affibody, a small triple-helix-domain protein derived from staphylococcal protein A, to create CAR-NK-92MI cells. While this CAR-NK therapy required irradiation attenuation because NK-92 is a continuously growing cell line, combination treatment with HER2-specific CAR-NK cells and doxorubicin loaded nanoparticles increased the anti-tumor potency of CAR-NK cells in preclinical models of HER2-postiive breast cancer models.

## Cytokine modulation and armored CAR-NK constructs

Beyond receptor design, multiple contributions emphasize the role of cytokines and intrinsic messenger signals in enhancing CAR-NK cell activity, particularly in immune-hostile TME that dampens NK function. Among these, IL-15 cytokine-armored CAR-NK cells highlights both opportunity and caution in a Perspective article from Baughan et al. IL-15 armoring, which enables autocrine growth and survival signaling, can bolster CAR-NK expansion, persistence, and cytotoxicity. However, preclinical studies using immunodeficient mouse models reveal toxicity associated with unchecked CAR-NK expansion due to rampant proliferation in the absence of host regulatory immune cells, usually independent of classical cytokine release syndrome. The authors propose that humanized models or integrated kill switches could mitigate this risk, a key consideration as armored CAR-NK cells advance toward clinical evaluation.

Collectively, it is emphasized that cytokine support, whether through engineered expression (e.g., IL-15), combined cytokine cocktails, or synthetic biology circuits, can amplify NK persistence and function, but must be balanced with safety controls. These insights will be critically important as the field moves toward clinically deployable armored CAR-NK designs.

## Toward solid tumor efficacy: trafficking and microenvironmental challenges

Despite advances in the field, solid tumors remain a formidable challenge for CAR-based immunotherapy. Guo et al. review the existing studies to dissect mechanisms of tumor resistance and highlight engineering strategies to improve NK homing, migration, and gene-edited functionality. Enhancing chemokine receptor alignment and modifying inhibitory pathways are emphasized as means to increase tumor infiltration and persistence. Hou et al. places CAR-NK within a broader ecosystem of engineered innate and innate-like immune cells. The authors compare CAR-NK with CAR-macrophage (CAR-M) and CAR-γδ T platforms, proposing combinatorial and complementary strategies to overcome antigen heterogeneity, stromal barriers, and immunosuppressive TME. While CAR-NK cells could be engineered based on principles of NK cell cytotoxicity, Niedzielska et al. systematically review and discuss how CAR-invariant NKT (iNKT) cells integrate and leverage both innate and adaptive features, CD1d recognition, NK/iNKT-specific receptor signaling, and CAR specificity simultaneously. Their capacity to remodel the TME and activate endogenous immunity positions CAR-iNKT cells as a powerful allogeneic alternative platform, closely aligned with the conceptual advantages of CAR-NK cells.

## Manufacturing and expansion: toward scalable platforms

Clinical translation requires scalable and safe manufacturing solutions. Traditional feeder cell systems raise safety and regulatory concerns. The authors in Lee et al. review cytokine-based, antibody-mediated, nanoparticle-assisted, and hydrogel-based feeder-free approaches, highlighting nanoparticle strategies as particularly promising for standardized, scalable expansion. This theme is further expanded in Yu et al.‘s article, which surveys nanotechnology applications in NK therapy like nanoparticle-assisted immune modulation and TME remodeling. By integrating nanomaterials with CAR-NK platforms, this approach may enhance trafficking, persistence, and local activation. Together, these contributions underscore that engineering innovation must extend beyond receptor design to encompass manufacturing, delivery, and microenvironment modulation.

## Future directions

Collectively, these studies redefine CAR-NK engineering as a distinct discipline within synthetic immunology. Rather than serving as a derivative of CAR-T therapy, CAR-NK research is developing its own conceptual framework rooted in innate immune biology, technological innovation, and translational foresight. As these advances converge, the vision of safe, effective, and widely accessible off-the-shelf cellular immunotherapies for both hematologic and solid malignancies become increasingly attainable.

